# Supplementary material for: Impaired Efflux of the Siderophore Enterobactin Induces Envelope Stress in Escherichia coli
Source: Front Microbiol. 2019 Dec 6;10:2776. doi: 10.3389/fmicb.2019.02776 (PMC6908949; doi:10.3389/fmicb.2019.02776)
Supplement: Supplementary file 1 [file Data_Sheet_1.docx]

**Supplemental material for:**

**Impaired Efflux of the Siderophore Enterobactin Induces Envelope Stress In *Escherichia coli***

Randi L. Guest^1,#^, Emily A. Court^1^, Jayne L. Waldon^1^, Kiersten A. Schock^1^, and Tracy L. Raivio^1^*

^1^Tracy L. Raivio, Department of Biological Sciences, University of Alberta, Edmonton, Alberta, Canada T6G 2E9

#Present address:

Department of Molecular Biology, Lewis Thomas Lab, Princeton University, Princeton, NJ, USA 08544

*Corresponding author:

Phone: +1 (780) 492-3491

Fax: +1 (780) 492-9234

E-mail: traivio@ualberta.ca

**Materials and Methods**

**Sequence alignment**

The DNA sequence of the *entCEBA* promoter region in enteropathogenic *E. coli* strain E2348/69 and *E. coli* K-12 strain MC4100 obtained from the cloned pJW15-P*entCEBA*_EPEC_ and pJW15-P*entCEBA*_K-12_ reporter plasmids. DNA sequencing was performed by the Molecular Biology Services Unit at the University of Alberta. The DNA sequence of the insert for the pJW15-P*entCEBA*_EPEC_ and pJW15-P*entCEBA*_K-12_ was compared to the published genome of MG1655 and E2348/69, respectively, and was found to be 100% identical (data not shown). DNA sequences were aligned using Multialin (<http://multalin.toulouse.inra.fr/multalin>).

**Figures and Tables**

| Table S1. Bacterial strains and plasmids used in this study | | |
| --- | --- | --- |
| **Strain or plasmid** | **Description** | **Source or reference** |
|  | | |
| *Strains* | | |
| MC4100 | F’ *traD36 lacI^q^ Δ(lacZ*)*M15 proA^+^B^+^/* e14 (McrA^-^) *Δ(lac-proAB*) *thi gyrA96* (Nal^r^) *endA1 hsdR17*(r_k_^-^ m_k_^+^) *relA1 supE44 recA1*; Str^R^ | (Casadaban, 1976) |
| TR10 | MC4100 *cpxA24*; Amk^R^ | (Raivio *et al.*, 1999) |
| TR50 | MC4100 λRS88[*cpxP’-lacZ^+^*]; Str^R^ | (Raivio and Silhavy, 1997) |
| TR51 | MC4100 *cpxR::spc*; Spc^R^ | (Raivio *et al.*, 1999) |
| JW0585 | BW25113 Δ*entC::kan*; Kan^R^ | (Baba *et al.*, 2006) |
| JW1095 | BW25113 Δ*ndh::kan*; Kan^R^ | (Baba *et al.*, 2006) |
| JW5503 | BW25113 Δ*tolC::kan*; Kan^R^ | (Baba *et al.*, 2006) |
| DY378 | W3110 λcI857 Δ(*cro-bioA*) | (Yu *et al.*, 2000) |
| E2348/69 | Prototypical EPEC O127:H6 strain; Str^R^ | (Levine *et al.*, 1978) |
| ALN195 | E2348/69 *cpxA24*; Str^R^ Amk^R^ | (MacRitchie *et al.*, 2008) |
| RG222 | E2348/69 Δ*cpxRA*; Str^R^ | (Guest *et al.*, 2017) |
| EC3 | TR50 Δ*tolC*; Str^R^ | This study |
| EC4 | TR50 Δ*tolC* Δ*entC::kan*; Kan^R^ | This study |
| RG244 | TR50 Δ*entC::kan*; Kan^R^ | This study |
| RG249 | TR50 Δ*ndh::kan*; Kan^R^ | This study |
| RG250 | TR50 Δ*tolC* Δ*ndh::kan*; Kan^R^ | This study |
| RG280 | TR50 Δ*cpxA::kan*; Kan^R^ | This study |
| RG281 | TR50 Δ*tolC* Δ*cpxA::kan*; Kan^R^ | This study |
| RG383 | DY378 Δ*nuoABCDEFGHIJKLMN::kan*; Kan^R^ | This study |
| RG392 | TR50 Δ*nuoABCDEFGHIJKLMN::kan*; Kan^R^ | This study |
| RG397 | DY378 Δ*cyoABCDE::kan*; Kan^R^ | This study |
| RG436 | TR50 Δ*cyoABCDE::kan*; Kan^R^ | This study |
| RG479 | TR50 Δ*tolC* Δ*nuoABCDEFGHIJKLMN::kan*; Kan^R^ | This study |
| RG480 | TR50 Δ*tolC* Δ*cyoABCDE::kan*; Kan^R^ | This study |
|  | | |
| *Plasmids* | | |
| pFLP2 | Broad host-range plasmid expressing the FLP recombinase from a temperature sensitive promoter; Amp^R^ | (Hoang *et al.*, 1998) |
| pJW15- P*entCEBA_K-12_* | pJW15 luminescence reporter plasmid containing the MC4100 *entCEBA* promoter; Kan^R^ | This study |
| pJW15-P*entCEBA*_EPEC_ | pJW15 luminescence reporter plasmid containing the E2348/69 *entCEBA* promoter; Kan^R^ | This study |

| **Table S2. Oligonucleotide primers used in this study** | |
| --- | --- |
|  |  |
| **Primer name** | **Sequence*** |
| PentFEcoRI | 5’-TTTTGAATTCCTGAACTGCGGCTATTCCTG-3’ |
| PentRBamHI | 5’-TTTTGGATCCTACTTCCTCAGCCAGTGACG-3’ |
| K12-cyoKOF | 5’-CCACACACTTTAAACGCCACCAGATCCCGTGGAATTGAGG TCGTTAAATGATTCCGGGGATCCGTC-3’ |
| K12-cyoKOR | 5’-CGTAGCACCTTTTTAATAGAGAGGTTTTGTTACCACACAGCA GCCAGCAGTGTAGGCTGGAGCTGC-3’ |
| K12nuoKOF | 5’-CTGCCGTGAAGAGCAGTGAATCTGGCGCTACTTTTGATGAGT AAGCAATGATTCCGGGGATCCGTC-3’ |
| K12nuoKOR | 5’-GGCGGCTTTCTGACTTACAAAGTAACAGATTACATCAGCGGC ATTGCCAATGTAGGCTGGAGCTGC-3’ |
|  | |
| *Underlining denotes a restriction enzyme sequence (BamHI: GGATCC; EcoRI: GAATTC) | |

| **** |
| --- |
|  |

**Figure S1: Enterobactin accumulation activates the Cpx response in the *tolC* mutant.** After growth overnight in LB broth, bacteria were washed and resuspended in phosphate buffered saline. 10μL of culture was spotted onto M9 minimal medium agar containing 0.4% glucose and grown at 37^o^C for 24 hours. Bacteria were scraped off the agar surface using inoculating loops and resuspended in 1 x Z buffer. *cpxP-lacZ* activity was measured as described in the materials and methods section of the main text. Data represent the means and standard deviations of three biological replicates. Asterisks indicate a statistically significant difference in *cpxP-lacZ* activity between the indicated strains (****, *P* ≤ 0.0001 [two-way ANOVA with Sidak’s post-hoc test]).

| **A.** | **** |
| --- | --- |
|  |  |
| **B.** | **** |

**Figure S2: Comparison of *entCEBA* expression in MC4100 and EPEC.** (A) Activity of the enteropathogenic *E. coli* (EPEC) *entCEBA-lux* reporter and the MC400 *entCEBA-lux* reporter in wildtype EPEC or wildtype MC4100. Bacteria were grown overnight in LB broth. The following day, bacteria were subcultured into M9 minimal medium containing 0.4% glucose, 5.34mM isoleucine, and 6.53mM valine at a dilution factor of 1:100. *entCEBA-lux* expression was measured after 8 hours of growth at 30^o^C as described in the materials and methods section of the main text. Data represent the means and standard deviations of five biological replicates. Asterisks indicate a statistically significant difference between the indicated strains (****, *P* ≤ 0.0001; ***, *P* ≤ 0.001 [one-way ANOVA with Sidak’s post-hoc test]). NS indicates no statistically significant difference in *entCEBA-lux* reporter activity. (B) Alignment of the *entCEBA* promoter region between MC4100 and E2348/69. DNA sequence of the *entCEBA* promoter DNA was determined by sequencing the insert of the pJW15-P*entCEBA*_K-12_ and pJW15-P*entCEBA*_EPEC_ plasmids. The DNA sequences were aligned using Multialin (<http://multalin.toulouse.inra.fr/multalin>). Bolded sequences represent the -35 box (TTGACA), the -10 box (TAGGTT), and the start codon (ATG), which were identified using the Ecocyc database (http://ecocyc.org) (Keseler *et al.*, 2011). Red sequences denote single base pair changes; -, absence of a base pair.

**References**

Baba, T., Ara, T., Hasegawa, M., Takai, Y., Okumura, Y., Baba, M., et al. (2006). Construction of Escherichia coli K-12 in-frame, single-gene knockout mutants: the Keio collection. *Mol. Syst. Biol.* 2, 2006.0008. doi:10.1038/msb4100050.

Casadaban, M. J. (1976). Transposition and fusion of the lac genes to selected promoters in Escherichia coli using bacteriophage lambda and Mu. *J. Mol. Biol.* 104, 541–555.

Guest, R. L., Wang, J., Wong, J. L., and Raivio, T. L. (2017). A Bacterial Stress Response Regulates Expression of Respiratory Protein Complexes to Control Envelope Stress Adaptation. *J. Bacteriol.*, JB.00153–17. doi:10.1128/JB.00153-17.

Hoang, T. T., Karkhoff-Schweizer, R. R., Kutchma, A. J., and Schweizer, H. P. (1998). A broad-host-range Flp-FRT recombination system for site-specific excision of chromosomally-located DNA sequences: application for isolation of unmarked Pseudomonas aeruginosa mutants. *Gene* 212, 77–86.

Keseler, I. M., Collado-Vides, J., Santos-Zavaleta, A., Peralta-Gil, M., Gama-Castro, S., Muñiz-Rascado, L., et al. (2011). EcoCyc: a comprehensive database of Escherichia coli biology. *Nucleic Acids Res.* 39, D583–90. doi:10.1093/nar/gkq1143.

Levine, M. M., Bergquist, E. J., Nalin, D. R., Waterman, D. H., Hornick, R. B., Young, C. R., et al. (1978). Escherichia coli strains that cause diarrhoea but do not produce heat-labile or heat-stable enterotoxins and are non-invasive. *Lancet* 1, 1119–1122.

MacRitchie, D. M., Ward, J. D., Nevesinjac, A. Z., and Raivio, T. L. (2008). Activation of the Cpx Envelope Stress Response Down-Regulates Expression of Several Locus of Enterocyte Effacement-Encoded Genes in Enteropathogenic Escherichia coli. *Infection and Immunity* 76, 1465–1475. doi:10.1128/IAI.01265-07.

Raivio, T. L., and Silhavy, T. J. (1997). Transduction of envelope stress in Escherichia coli by the Cpx two-component system. *J. Bacteriol.* 179, 7724–7733.

Raivio, T. L., Popkin, D. L., and Silhavy, T. J. (1999). The Cpx envelope stress response is controlled by amplification and feedback inhibition. *J. Bacteriol.* 181, 5263–5272.

Yu, D., Ellis, H. M., Lee, E. C., Jenkins, N. A., Copeland, N. G., and Court, D. L. (2000). An efficient recombination system for chromosome engineering in Escherichia coli. *Proc. Natl. Acad. Sci. U.S.A.* 97, 5978–5983. doi:10.1073/pnas.100127597.
